# The Combination of Rhodosin and MMF Prolongs Cardiac Allograft Survival by Inhibiting DC Maturation by Promoting Mitochondrial Fusion

**DOI:** 10.1155/2022/7260305

**Published:** 2022-07-09

**Authors:** Yanjia Che, Yuanyang Chen, Zhiwei Wang, Sihao Zheng, Kai Xing, Shun Yuan, Xiaohan Zhong

**Affiliations:** ^1^Department of Cardiovascular Surgery, Renmin Hospital of Wuhan University, Wuhan 430060, China; ^2^Cardiovascular Surgery Laboratory, Renmin Hospital of Wuhan University, Wuhan 430060, China; ^3^Central Laboratory, Renmin Hospital of Wuhan University, Wuhan 430060, China

## Abstract

Despite being the gold-standard treatment for end-stage heart disease, heart transplantation is associated with acute cardiac rejection within 1 year of transplantation. The continuous application of immunosuppressants may cause side effects such as hepatic and renal toxicity, infection, and malignancy. Developing new pharmaceutical strategies to alleviate acute rejection after heart transplantation effectively and safely is of critical importance. In this study, we performed a murine model of MHC-full mismatch cardiac transplantation and showed that the combination of Rhodosin (Rho) and mycophenolate mofetil (MMF) could prevent acute rejection and oxidative stress injury and prolong the survival time of murine heart transplants. The use of Rho plus MMF in allografts improved the balance of Tregs/Teff cells, which had a protective effect on allotransplantation. We also isolated bone marrow-derived dendritic cells (BMDCs) and determined that Rho inhibited DC maturation by promoting mitochondrial fusion mainly through the mitochondrial fusion-related protein MFN1. Herein, we demonstrated that Rho, an active ingredient isolated from the plant *Rhodiola rosea* with antioxidant and anti-inflammatory activities, could efficiently alleviate acute rejection and significantly prolong murine heart allograft survival when used with a low dose of MMF. More importantly, we found that Rho restrained DC maturation by promoting mitochondrial fusion and decreasing reactive oxygen species (ROS) levels, which then alleviated acute rejection in murine cardiac transplantation. Interestingly, as a novel immunosuppressant, Rho has almost no side effects compared with other traditional immunosuppressants. Taken together, these results suggest that Rho has good clinical auxiliary applications as an effective immunosuppressant and antioxidant, and this study provides an efficient strategy to overcome the side effects of immunosuppressive agents that are currently used in organ transplantation.

## 1. Introduction

End-stage heart disease is responsible for millions of deaths worldwide each year [1]. Heart transplantation is the most effective treatment for end-stage heart diseases [2]. Although significant progress in heart transplantation has been made in recent decades, recipient rejection after transplantation is not entirely controlled, and long-term administration of immunosuppressive drugs is required to avoid cardiac allograft rejection. There can be adverse effects, such as infection, renal toxicity, metabolic diseases, and malignancy, and novel immunosuppressive agents with minimal side effects are still in high demand [3]. Allograft survival depends on the successful induction of immune tolerance after transplantation. In particular, tolerogenic dendritic cells (tol-DCs) play important effects in maintaining immune tolerance [4]. Thus, new immunosuppressants with better curative effects and fewer side effects that can induce tol-DCs are urgently needed in heart transplantation.

DCs form a bridge connecting the innate and adaptive immune systems [5]. DCs can induce naïve T cells to differentiate into different phenotypes, including CD4^+^/8^+^ T cells and regulatory T cells (Tregs) [6]. For the purpose of improving transplant survival and reducing dependency on immunosuppressants, numerous approaches have been used to inhibit DC maturation to improve heart transplantation patient survival and quality of life. Thus, tol-DCs with low levels of MHC II, CD80, and CD86 induce alloantigen-specific T cell hyporesponsiveness and the expansion of Tregs and promote the secretion of anti-inflammatory cytokines to prolong the survival time of grafted organs [5]. Since MHC II and costimulatory molecules are highly expressed on the surface of mature DCs, mature DCs play vital roles in mediating the activation and proliferation of T cells, which act as effector cells in acute rejection. Inhibiting DC maturation and then restraining T cell activation is a good strategy for alleviating acute rejection after organ transplantation in patients [4].

Rhodosin (Rho) is the main bioactive extract from *Rhodiola rosea*. Rho has attracted much attention because of its potential effects on cardiovascular diseases, including endothelial cell injury [7–10], atherosclerotic plaque formation [11–13], myocardial injury [14–17], and pulmonary hypertension [18–20]. It has been confirmed that Rho protects against ischemia-reperfusion injury due to its anti-inflammatory and antiapoptotic effects [21]. Importantly, Rho can also upregulate CD4^+^CD25^+^Foxp3^+^ Tregs in autoimmune encephalomyelitis [22]. However, it is unknown whether Rho is beneficial in heart allograft rejection and immune tolerance. MMF is a traditional immunosuppressant that is widely used in posttransplantation patients and for other conditions, but the use of this compound in large quantities and for long periods of time can induce strong side effects, such as gastrointestinal dysfunction and increased likelihood of infection; thus, we tried to use natural botanicals in combination with MMF to reduce the required dose of MMF and achieve long-term tolerance in patients. This study is therefore designed to comprehensively investigate the synergetic effects of Rho and MMF in preventing allograft rejection via the maintenance of innate and adaptive immunosuppression.

Mitochondria are highly mobile organelles that have many physiological functions, including affecting the immune system. Mitochondrial dynamics consist of mitochondrial fusion and fission. Studies have shown that mitochondrial dynamics are related to the types of T cells and the polarization of macrophages [23–25]. Mitochondrial fusion can take place in the mitochondrial outer membrane (MOM) and mitochondrial inner membrane (MIM). MOM fusion is mediated by mitofusin 1 (MFN1) and mitofusin 2 (MFN2). Fusion of the MIM is mediated by the optic atrophy 1 (OPA1) protein. Mitochondrial fission is the division of one mitochondrion into two. Mitochondrial fission is mainly mediated by dynamic-related protein 1 (Drp1), which needs to anchor adaptor proteins such as fission protein 1 (Fis1) to play a critical role in fission. We determined the protein expression of these mitochondrial dynamics-related proteins in bone marrow-derived dendritic cells (BMDCs) after Rho treatment to examine mitochondrial dynamics.

In this study, we hypothesized that the combination of Rho and MMF could promote the mitochondrial fusion and then inhibit the maturation of DCs; based on this mechanism, combination administration prolonged graft survival and clearly ameliorated immune cell infiltration and myocardial apoptosis. To explore this, using the fully MHC II-mismatch acute heart transplantation, we test the effects of combination administration of MMMF and Rho in allografts. We found that the combination of Rho and MMF increased the percentage of CD4^+^CD25^+^Foxp3^+^ Tregs and reduced the numbers of mature DC and CD4^+^/CD8^+^ T cell posttransplantation. In particular, the percentages of T effector (Teff)/memory cells (CD44^hi^CD62L^low^) among CD4^+^ T cells in the spleen and draining lymph nodes (LNs) were significantly reduced in Rho-treated groups. For further insight into the mechanism by which Rho inhibits DCs, we determined that the effect of Rho depended on the promotion of mitochondrial fusion. To the best of our knowledge, there is no study that has shown that Rho or even *Rhodiola* has a protective role in preventing acute rejection after heart transplantation. In addition, the effects of the mitochondrial dynamics of DCs on the induction of immune tolerance have never been reported. Thus, we demonstrated that Rho was a novel immunosuppressant that regulates the mitochondrial dynamics of DCs and exerts cardiovascular protective effects on heart allotransplantation.

## 2. Materials and Methods

### 2.1. Materials

Rho (purity > 98%) was supplied by Aladdin Chemistry Co., Ltd. Male C57BL/6 (H-2^b^) and BALB/c (H-2^d^) mice (7-8 weeks old, 20-25 g) were purchased from Beijing Vital River Laboratory Animal Technology Co., Ltd. All mice were housed in a sterile environment and were used and cared for in accordance with institutional guidelines. All protocols used in this study were reviewed and approved by the Animal Ethics Committee of Renmin Hospital of Wuhan University (IACUC Issue No. 20201107).

### 2.2. Heart Transplantation

Donor hearts were collected from 8-week-old BALB/c (H-2^d^) mice, and the recipients were 8-week-old C57BL/6 mice (H-2^b^). Fully vascularized heterotopic heart transplantation was performed as described previously [26]. Prior to the start of the study, the authors had undergone extensive practice with the heart transplantation model and had become very proficient in performing this procedure. According to our experimental statistics, the cold ischemia time during transplantation was maintained at 8 ± 2.3 (*n* = 120) minutes, and due to extensive practice, we were able to ensure that the cold ischemia time remained consistent for each model mouse within 2.3 minutes. Briefly, the donor aorta and pulmonary artery were anastomosed to the recipient carotid artery and vena jugularis externa via the cuff technique [27]. The success rate of the murine heart transplant model is 60%. A total of 80 mice were used, 40 of which were C57BL/6 recipients and 40 of which were BALB/c donors. Graft surveillance was monitored daily by recipient cervical palpation and inspection. Five days after the operation, the grafts and recipient spleens and draining LNs were removed and analyzed.

### 2.3. Drug Administration

Blindly dividing the recipients into four groups: control group (intraperitoneal injection (i.p.) saline), Rho group (i.p. 50 mg/kg Rho), MMF group (i.p. 160 mg/kg), and Rho + MMF (i.p. 50 mg/kg Rho and 80 mg/kg MMF). The animals were treated daily for 5 consecutive days after transplantation until graft rejection/sample collection. Rho was dissolved in saline. The control group was administered saline only.

### 2.4. Culture of Murine BMDCs

Murine BMDCs from male C57BL/6 mice (8 weeks old, 20-25 g) were propagated. In brief, bone marrow cells were flushed out from the femurs and tibias. After the red cells were lysed, the cells were cultured in RPMI 1640 medium containing 10 ng/ml recombinant granulocyte macrophage colony-stimulating factor (GM-CSF) and 10 ng/ml interleukin-4 (IL-4) at 37°C with 5% CO_2_ for 7 days in a 12-well plate. On day 6, immature DCs were pretreated with Rho or PBS for 12 h. The cells were subsequently stimulated with 100 ng/ml lipopolysaccharide (LPS) to induce DC maturation. The medium was refreshed every 3 days, and at the 7^th^ day of incubation, the cells were harvested and analyzed by flow cytometry (CytoFlex, Beckman Coulter Biotechnology Co., Ltd). Three mice are required for BMDCs cell extraction.

### 2.5. Flow Cytometry

Draining LNs and spleens were harvested from recipient mice. After homogenization and red cell lysis, the cells were stained with anti-mouse CD4-FITC (clone H129.19), anti-mouse CD8*α*-PE (clone 53-6.7), anti-mouse CD25-PE (clone PC-61), anti-mouse CD11c-PE (clone HL3), anti-mouse CD44-PE (clone IM7), anti-mouse-CD62L-APC (clone MEL-14), anti-mouse MHC II-FITC (clone AF6-120.1), anti-mouse CD86-FITC (clone GL1), and anti-mouse-CD80-FITC (clone 16-10A1). The protocol for Treg analysis is described in our previous study [28]. The data were analyzed by the FlowJo software V10. The maturation of BMDCs was investigated by staining the cells with duplicate fluorescent-labeled antibodies and anti-mouse-CD11c-PE (clone HL3), anti-mouse MHC II-FITC (clone AF6-120.1), anti-mouse CD86-FITC (clone GL1), and anti-mouse-CD80-FITC (clone 16-10A1), after which the cells were analyzed by flow cytometry.

### 2.6. Real-Time PCR

RNA was extracted from BMDCs using a UNIQ-10 Column TRIzol Total RNA Isolation Kit (Sangon Biotech, China) according to the manufacturer's protocols. Next, a reverse transcription kit (Yeasen, #11119Es60, China) was used in a Bio-Rad PCR system (Bio-Rad, USA). The CFX96 Real-Time PCR System with specific primers and software (Bio-Rad, Hercules, CA, USA) was used to perform the real-time quantitative PCR. For RNA analysis, the levels of IL-1*β*, IL-6, IL-10, and IL-12 were quantified.

The primers used for RT–PCR were as follows:

IL-1*β* (forward: 5′- GAAATGCCACCTTTTGACAGTG-3′, reverse: 5′-TGGATGCTCTCATCAGGACAG-3′), IL-6 (forward: 5′-CTGCAAGAGACTTCCATCCAG-3′, reverse: 5′-AGTGGTATAGACAGGTCTGTTGG-3′), IL-10 (forward: 5′-CTTACTGACTGGCATGAGGATCA-3′, reverse: 5′-GCAGCTCTAGGAGCATGTGG-3′), and IL-12 (forward: 5′-GTCCTCAGAAGCTAACCATCTCC-3′,reverse: 5′-CCAGAGCCTATGACTCCATGTC-3′).

### 2.7. Mixed Lymphocyte Reaction

The C57BL/6 splenic cells were made into single cell suspensions. And the cells were stained with anti-CD4 FITC antibodies for 30 minutes. Then the cells were sorted by the S^3^TM Cell Sorter (Bio-Rad Laboratories, Inc.). The T cells and pretreated DCs or unpretreated DCs were seeded together in 96-well plates. After coincubation for 72 h, cell proliferation was measured with the CFDA SE Cell Proliferation Assay and Tracking Kit (Beyotime, #C0051, China) according to the manufacturer's protocol.

### 2.8. Western Blotting

To gain insight into the molecular mechanism of DCs after being stimulated, western blotting was used to assess the expression of DRP1, OPA1, MFN1, MFN2, phospho-DRP1 (Ser637), phospho-DRP1 (Ser616), Fis1, and GAPDH in immature DCs, mature DCs, and Rho-treated DCs. Briefly, total proteins were obtained after the cells were exposed to LPS, and protein concentration was determined by a BCA kit. Proteins were separated by gel electrophoresis and then transferred to PVDF membranes. After being blocked with 5% milk powder in PBST for 1.5 h, the PVDF membranes were incubated with DRP1 rabbit polyclonal antibodies (Beyotime, #AF6735, China), OPA1 rabbit polyclonal antibodies (Beyotime, #AF7653, China), MFN1 rabbit polyclonal antibodies (Beyotime, #AF7461, China), MFN2 rabbit polyclonal antibodies (Beyotime, #AF7473, China), phospho-DRP1 (Ser637) rabbit polyclonal antibodies (Beyotime, #AF5791, China), and phospho-DRP1 (Ser616) antibodies (Affinity Bioscience, #AF8460, China). The protein levels were quantified using scanning densitometry (GS-710 imaging). The data were obtained in triplicate for independent experiments.

### 2.9. Histopathological Staining

On day 5 after the operation, the heart grafts were harvested. After paraformaldehyde fixation and paraffin-embedding, the heart grafts were cut into paraffin sections, stained with HE, and observed by light microscopy (BX51, Olympus, Japan). The standards published by the International Society for Heart and Lung Transplantation were used to evaluate the grades of the rejection response [29]. For immunofluorescence staining, the sections were dewaxed, restored by microwave heating, blocked with 5% goat serum (Beyotime Bio, #C0265, China), and then incubated with anti-CD4 (1 : 100, Santa Cruz, #sc-20079, China) and anti-CD8 (1 : 100, Santa Cruz, #sc-1177, China) overnight at 4°C. The next day, the sections were incubated with CY3-labeled goat anti-mouse secondary antibodies (1 : 50, Servicebio, #GB22301, China) and subsequently stained with 4′,6-diamidino-2-phenylindole (DAPI, 2 *μ*g/ml; Servicebio, #G1012-10ML, China) for five minutes. The images were acquired by fluorescence microscopy (Olympus BX6 with a DP72 Camera, Japan) and then analyzed by the ImageJ software.

### 2.10. Mitochondrial ROS Assay

BMDCs were incubated with medium containing 100 ng/ml LPS (with or without 5 *μ*M Rho) for 12 h, and the cells were stained with 5 *μ*M DHE (Yeason, #50102ES02, China) for 30 minutes to detect reactive oxygen species (ROS) production. And meanwhile, the cells were stained with 2 ml 5 *μ*M MitoSOX Red Mitochondrial Superoxide Indicator (Yeason, #40778ES50, China) to detect mitochondrial reactive oxygen species (MitoROS) production. Then, the stained cells were analyzed with a Beckman Coulter instrument (Life Sciences).

### 2.11. MFN1 siRNA Transfection

BMDCs were incubated in FBS-free medium for 2 hours after 6 days of culture. Then, the cells were transfected with MFN1 siRNA (sense, 5′-GACGACCCGTGCGAAAGA-3′ and antisense, 5′-AGCTTCTCGGTTGCATAGGGGACA-3′) or nonspecific control siRNA (sense, 5′-UUCUCCGAACGUGUCACGUTT-3′ and antisense, 5′-AAGCCUAGUUCAAAGAUGGTT-3′; RiboBio, Guangzhou, China) (2 *μ*l, 20 *μ*M) using 4 *μ*l of Lipofectamine 3000 reagent in 1640 medium with 10% FBS. After being transfected for 48 h, the efficiency of MFN1 silencing was determined by western blotting.

### 2.12. Statistical Analysis

The data are expressed as the mean ± standard error of the mean (SEM) and were analyzed with the GraphPad Prism 8 software. Kaplan–Meier curves were used to analyze survival in the different groups. Student's *t* test was used for two-group comparisons. A value of *P* < 0.05 was considered statistically significant. Graphs were generated using the GraphPad Prism software version 8.0.

## 3. Results

### 3.1. The Combination of Rho and MMF Prolongs Heart Allograft Survival

**Figure 1:**
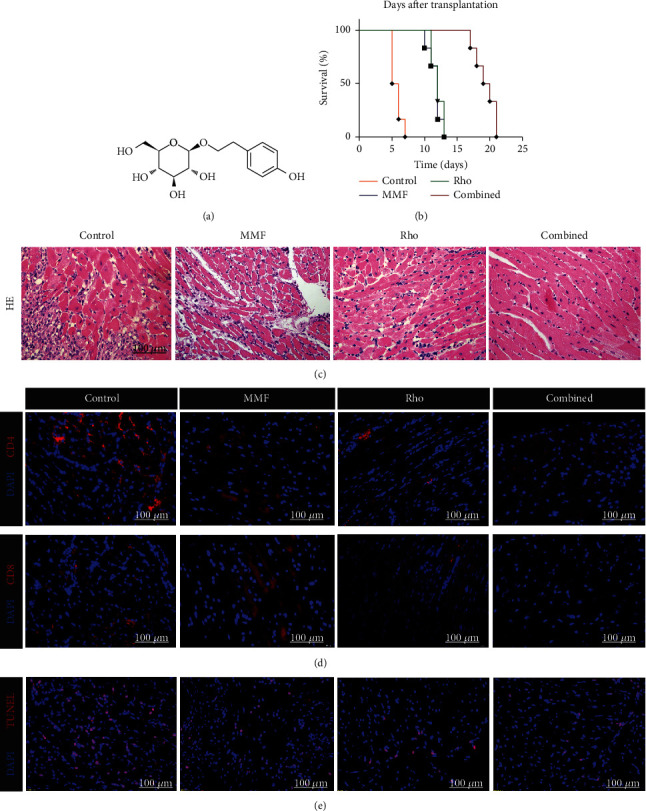
Combination of Rho and MMF prolongs cardiac allograft survival. Hearts from BALB/c donors were transplanted to C57BL/6 mice that were treated with Rho and combination of Rho and low dose of MMF after the operation 5 days. Cardiac allograft rejection was observed based on the cessation of palpable cardiac contraction (*n* = 6 mice per group). (a) Chemical structure of Rho. (b) The survival time of the cardiac grafts. In the combined treatment group, the mean survival time of the grafts was significantly longer (19.5 ± 0.56 days) than those of the control group (5.5 ± 0.81 days) (*n* = 6). (c) Hematoxylin-eosin (HE) staining on the cardiac allografts from control, MMF group, Rho-treated, and combination of Rho and low dose of MMF group. (d) The infiltration of CD4^+^ T cells and CD8^+^ T cells in transplanted hearts. (e) The immunofluorescence staining of TUNEL in allografts.

To evaluate the effects of combined treatment on heart transplantation outcomes, we transplanted BALB/c (H-2K^d^/I-A^d^) hearts into C57BL/6 (B6, H-2K^b^/I-A^b^) mice. This full MHC-mismatch mouse heart transplant model is a standard model for studying the acute rejection response to heart allografts. The chemical formula of Rho is shown in Figure 1(a). The cardiac allograft survival curve showed that both the Rho and MMF treatment groups had prolonged graft survival (Rho group: median survival 12.5 days and MMF group: median survival 12 days), and more importantly, the use of Rho and low dose of MMF prolonged graft survival even more significantly (combined group: median survival 19.5 days) (Figure 1(b)). We next assessed whether Rho plays a role in preventing immune infiltration in heart allografts, and we harvested the allografts 5 days after surgery. HE staining of explanted allografts on day 5 after transplantation showed diffuse lymphocyte infiltration with multifocal myocyte damage in the control group. In contrast, preserved cardiac architecture with reduced cellular infiltration was observed in the Rho group and combined group (Figure 1(c)) [30, 31]. To further observe immune infiltration in the grafts, immunofluorescence staining of CD4 and CD8 was performed. There were less CD4^+^ T cells and CD8^+^ T cells infiltration in the grafts in the Rho and combined groups than in the grafts in the control group (Figure 1(d)). Terminal deoxynucleotidyl transferase (TdT) dUTP nick-end labeling (TUNEL) is an assay to examine the localization of apoptotic DNA fragments. After transplantation, DNA fragmentation, as indicated by an increase in the number of TUNEL-positive nuclei, was observed in the control group compared with the Rho group and combined group. Treatment with Rho and a low dose of MMF significantly reduced myocardial apoptosis in the grafts (Figure 1(e)). We also evaluated the expressions of inflammatory factors in transplanted hearts; the results were shown in Supplementary Figure S1. Overall, these data indicate that the combination of Rho and MMF significantly mitigated acute rejection and myocardial apoptosis and improved graft survival.

### 3.2. The Combination of Rho and MMF Reduces the CD4^+^/CD8^+^ T Cell Populations and Increases the Percentage of Tregs In Vivo

**Figure 2:**
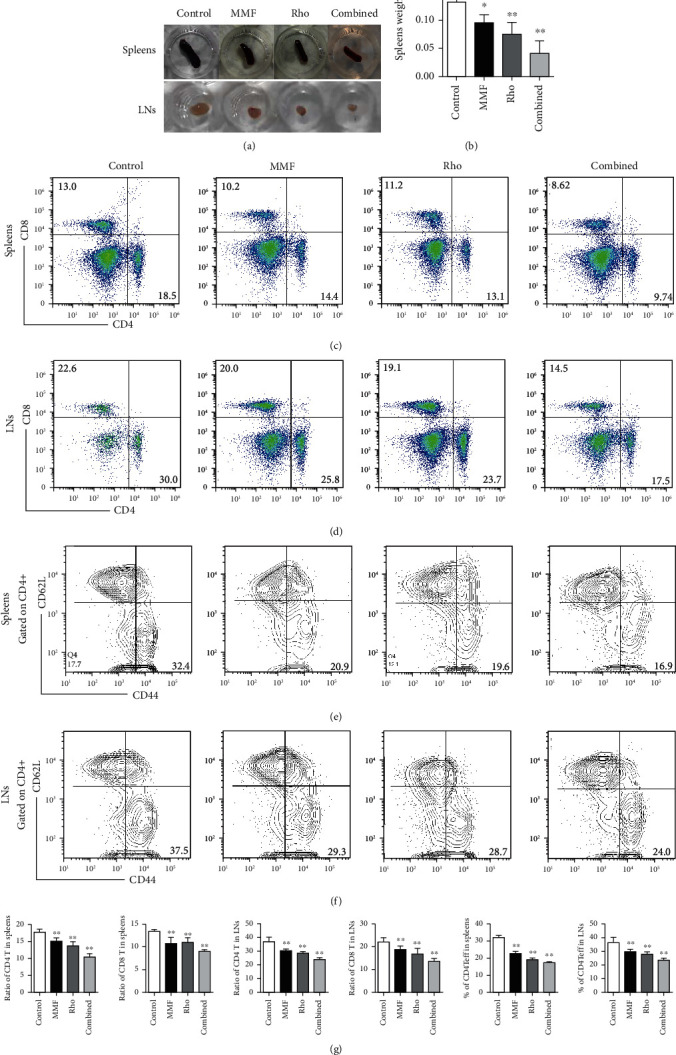
Effect of Rho and combination of Rho with low dose of MMF on the quantities of CD4^+^ and CD8^+^ T cells. The proportions of CD4^+^ and CD8^+^ T cells in the spleens and draining lymph nodes were determined by flow cytometry on day 5 after heart transplantation (*n* = 6 mice per group). (a) Macroscopic appearances of spleens and lymph nodes isolated from control group and MMF group, Rho group, and combination treatment group on day 5 after transplantation. (b) The weight (g) of spleens from different groups. (c) The proportions of CD4^+^ and CD8^+^ T cells in the spleens were determined by flow cytometry on day 5 after transplantation (*n* = 6 mice per group). (d) The proportions of CD4^+^ and CD8^+^ T cells in the draining lymph nodes were determined by flow cytometry on day 5 after transplantation (*n* = 6 mice per group). (e, f) Combination treatment mice showed low frequency of CD4^+^ T effector cells (CD4^+^CD44^hi^CD62L^low^) in spleens and draining lymph nodes. (g) The quantitative analysis of the CD4^+^ T, CD8^+^ T, CD4^+^ Teff cells in spleens and draining lymph nodes by GraphPad Prism 8.0. Data of histograms are presented as means ± SD from six independent experiments (^∗^*P* < 0.05, ^∗∗^*P* < 0.01, *n* = 6 mice per group versus the control group).

**Figure 3:**
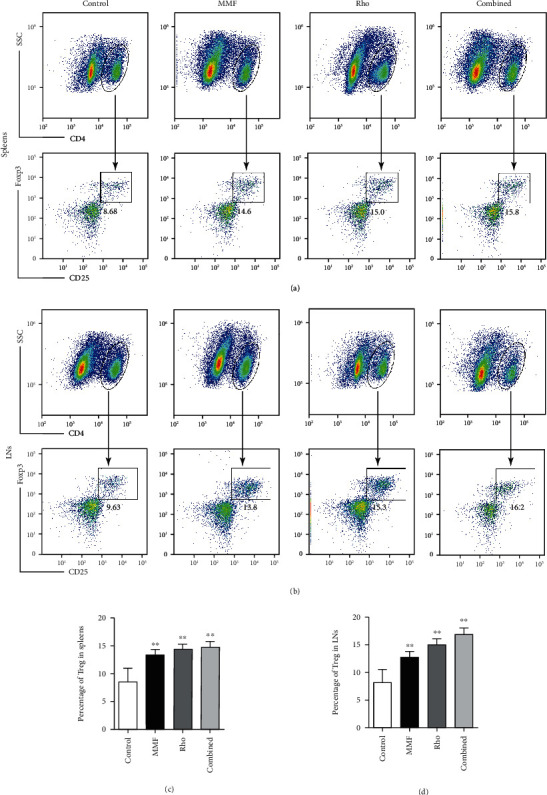
Rho and combination medication treatment increases the proportion of CD4^+^CD25^+^Foxp3^+^ T cells in CD4^+^T cells. (a) CD4 lymphocyte isolated from mice spleens treated with Rho and combination of Rho with low dose of MMF was shown to contain a higher proportion of Tregs (CD4^+^CD25^+^Foxp3^+^) than saline-treated Controls. (b) Percentage of Tregs (CD4^+^CD25^+^Foxp3^+^) in draining lymph nodes was significantly increased in Rho-treated and combination treatment mice in comparison with controls on isolation on day 5 after full MHC-mismatch cardiac transplantation (representative flow cytometry plots, pregated on CD4^+^ cells.). (c, d) The quantitative analysis of the Treg cells in CD4^+^ T cells in the spleens and draining lymph nodes by GraphPad Prism 8.0. Data of histograms are presented as means ± SD from six independent experiments (^∗^*P* < 0.05, ^∗∗^*P* < 0.01, *n* = 6 mice per group versus the control group).

Since we observed a significant effect of the combination of Rho and MMF on allografts, we next investigated the mechanism by which the combination of Rho and MMF conferred allograft protection via the immune system. The spleen and LNs are important peripheral immune organs that mainly mediate antigen presentation by antigen-presenting cells (APCs) to T cells [32]. T cells attack allografts after being stimulated by APCs. Therefore, we first examined the influence of Rho on the spleens and draining LNs (supraclavicular and axillary LNs) retrieved on day 5 after transplantation. Spleens and draining LNs from Rho-treated mice were smaller and weighed significantly less than those isolated from mice in the control groups (Figures 2(a) and 2(b)). CD4^+^ T cells play a dominant role and that CD8^+^ T cells has a supporting role in transplantation immunity [32]. To further investigate the effect of Rho and combined treatment on the proportions of CD4^+^ and CD8^+^ T cells in the spleen and draining LNs, flow cytometry was performed on day 5 after heart transplantation. Treatment with Rho evidently decreased the proportions of CD4^+^ T and CD8^+^ T cells in the spleens and draining LNs compared with those in the control group. Notably, the combination treatment had a synergistic effect in lowering the proportion of Teff cells (Figures 2(c) and 2(d)). Furthermore, the balance between Teff cells and Tregs influences the outcomes of heart allografts. The percentage of Teff/memory cells (CD44^hi^CD62L^low^) among CD4^+^ T cells in the spleens and LNs was significantly reduced in the Rho groups (Figures 2(e) and 2(f)). Tregs are able to suppress immune reactions and induce immune tolerance in alloimmunity [33]. Therefore, we examined the percentages of Tregs. The percentages of splenic Tregs (CD4^+^CD25^+^Foxp3^+^) were significantly increased in Rho-treated and combination-treated mice compared with control mice on day 5 after cardiac transplantation (Figures 3(a) and 3(c)). The same results were also observed in draining LNs (Figures 3(b) and 3(d)). We analyzed the ratio of CD4 Teff/Treg in spleens and LNs harvested from allotransplantation (Supplementary Figure S2). In summary, these results suggest that the combination of Rho and MMF can reduce the proportions of CD4^+^ or CD8^+^ T cells, thereby overcoming the challenge of immune injury mediated by T cells. Moreover, Rho and MMF treatment potentiated peripheral Tregs, which then contributed to the improvement of allograft survival.

### 3.3. The Combination of Rho and MMF Suppresses DC Maturation of DCs In Vivo

**Figure 4:**
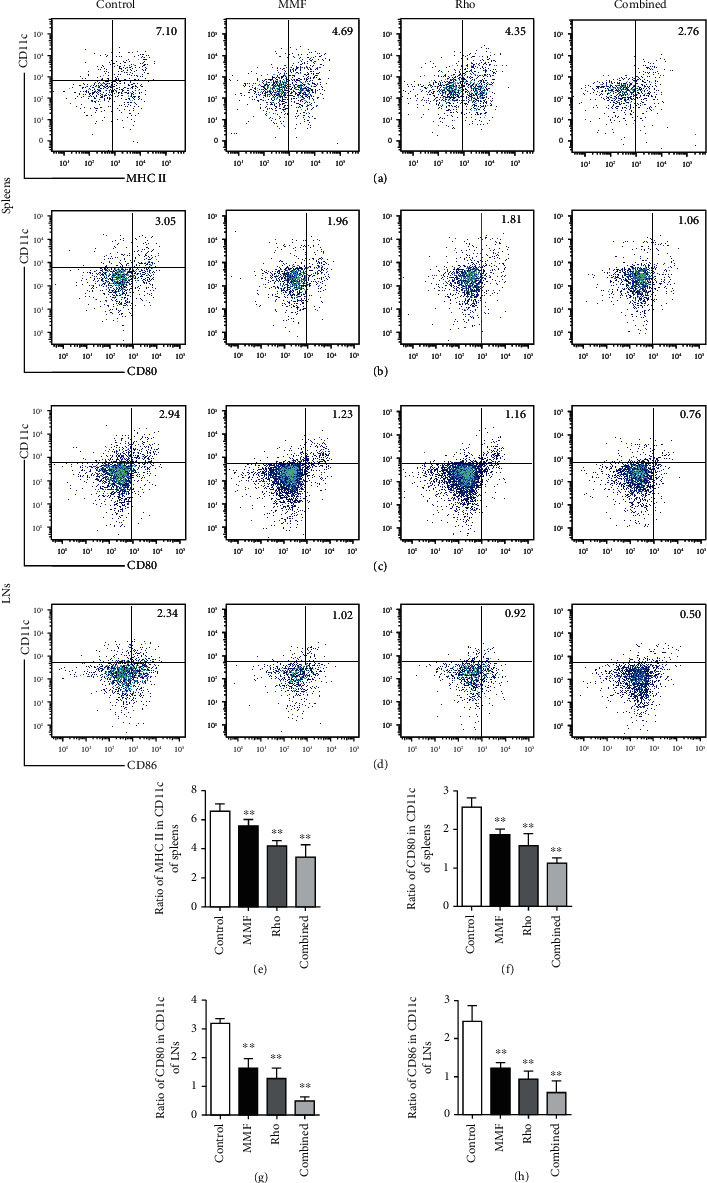
Rho and combination treatment interfere with DCs maturation after allotransplantation. Splenocytes and LN cells were collected from recipient mice or naïve mice and analyzed for the markers of DC maturation 5 d after heart allotransplantation. (a–d) The percentage of CD86^+^ MHC II^+^ CD80^+^ DCs within CD11c^+^ population was determined via the FACS analysis. Data of histograms are presented as means ± SD from six independent experiments (^∗^*P* < 0.05, ^∗∗^*P* < 0.01, *n* = 6 mice per group versus the control group). (e–h) The quantitative analysis of percentage of CD86^+^, MHC II^+^, and CD80^+^ DCs within CD11c^+^ population in spleens and draining lymph nodes by GraphPad Prism 8.0. Data of histograms are presented as means ± SD from six independent experiments (^∗^*P* < 0.05, ^∗∗^*P* < 0.01, *n* = 6 mice per group versus the control group).

Current therapies to prevent allograft rejection mainly depend on the use of drugs that inhibit nonspecific T cell activation and proliferation. Some novel strategies also target APCs, which stimulate T cell activation and expansion by costimulatory molecules. DCs play crucial roles in regulating the adaptive immune response by activating resting naïve T cells. These cells are pivotal in mounting an immune response through multiple mechanisms. Therefore, tol-DCs are aimed at making the immune response silent. Since our data demonstrated that Rho and combined treatment alleviated the immune response to heart allografts, we further hypothesized that Rho may have an immunosuppressive effect by restraining DC maturation. To evaluate the effect of Rho on DC maturation in vivo, splenocytes and draining LN cells were isolated five days after heart allotransplantation, and surface markers that are closely related to DC maturation were analyzed by flow cytometry. The Rho group and combined group showed significantly reduced percentages of CD86^+^, CD80^+^, and MHCII^+^ mature DCs within CD11c^+^ populations in both spleens and draining LNs (Figures 4(a)–4(h)). These data demonstrate that Rho can induce the generation of tol-DCs, which express low levels of MHC II, CD80, and CD86. This effect was more pronounced in the combination group.

### 3.4. Rho Inhibits DC Maturation, and Rho-Treated DCs Suppress T Cell Proliferation In Vitro

**Figure 5:**
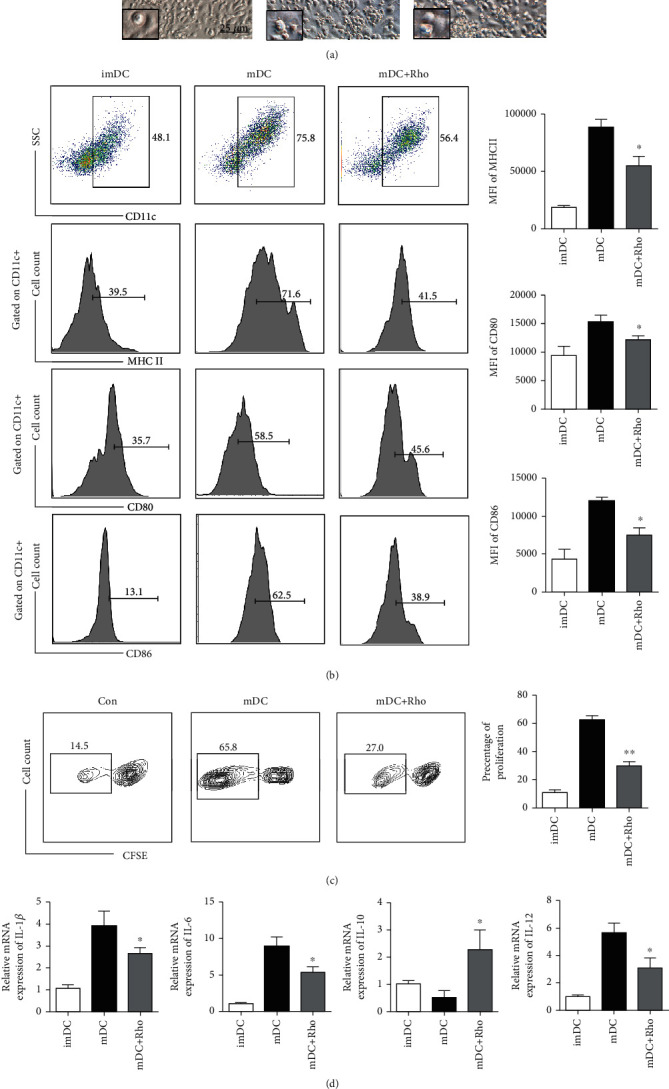
Rho treatment decreases the maturation of DCs, and Rho-treated DCs inhibited the proliferation of T lymphocytes in vitro. (a) Light microscopic analysis and SEM images of different groups of DCs. (b) The cell markers of DCs in CD11c population in different groups. (c) FACS-sorted CD4^+^ T cells from naïve C57BL/6 mice were labeled with CFSE and were treated with mature DC and Rho-treated DC for 72 h. (d) The secretion of cytokines including IL-1*β*, IL-6, IL-10, and IL-12 in different groups. The data are representative images or expressed as the means ± SD of three independent experiments. ^∗^*P* < 0.05, ^∗∗^*P* < 0.01 versus the mDC group.

After confirming that Rho downregulated MHC II, CD80, and CD86 expression in DCs in vivo, we evaluated the role of Rho in the maturation of BMDCs and Rho-treated DC-mediated attenuation of the alloimmune response in vitro. Mature DCs have a number of extended dendrites that interact with T cells and drive the activation of T cells. To examine the effect of Rho on the maturation of DCs in vitro, DCs were isolated from the bone marrow cavity and treated under different conditions, and then the morphological characteristics of DCs were observed. Compared with that in the LPS-stimulated control group, pretreatment with Rho (5 *μ*M) altered the morphology of mature DCs with dramatically decreased numbers of dendrites (Figure 5(a)). The scanning electron microscopy images (SEM) more graphically confirm the above description. And the clearer and larger images of SEM are provided in the Supplementary Figure S3.

To further investigate the maturation of BMDCs in the different groups, cell surface maturation markers (CD80, CD86, and MHC II) in the CD11c^+^ population were examined by flow cytometry. The results proved that Rho could inhibit the maturation of DCs (Figure 5(b)). And we supplemented the immunofluorescence analysis of MHC II expression on the surface of BMDC, which likewise confirmed the above findings (Supplementary Figure S4). Next, we investigated whether Rho-treated DCs could inhibit T lymphocyte proliferation. Lymphocytes were cultured with mature DCs and Rho-treated DCs for 72 h. We evaluated the ability of lymphocytes to proliferate by CFSE dilution. The result is shown in Figure 5(c). After 72 h of incubation, Rho-induced immature DCs significantly inhibited lymphocyte proliferation relative to those in the mature DC group. We next examined the cytokines that represent DC maturation. The levels of IL-1*β*, IL-6, IL-10, and IL-12 were measured by qPCR. The incubation of BMDCs with Rho led to reduced levels of IL-1*β*, IL-6, and IL-12 but increased IL-10 levels (Figure 5(d)). These results proved that Rho could restrain DC maturation in vitro. For excluding the direct effect of Rho on T cell proliferation and excitation by treating T cells with Rho in vitro. We have sorted the CD4^+^ T cells using S3™ Cell Sorter (Bio-Rad Laboratories, Inc.) (Supplementary Figure S5 (a)) and treated the T cell with Rho or without Rho directly. And we performed the CFSE experiments, the results were shown in the Supplementary Figure S5 (b). The results showed that Rho did not play direct effect on the proliferation of T cells. We also tested the expressions of IL-10 and IL-17 in CD4^+^ T cells treated with Rho-induced immature DCs by flow cytometry. The results showed that DC treated with Rho can increase the expression of IL-10 but decrease the expression of IL-17 in CD4^+^ T cells compared with treatment with mature DCs. The results were shown in Supplementary Figure S5 (c) and S5 (d). In summary, these data provide evidence that Rho can suppress the maturation of DCs and further inhibit the activation and proliferation of T cells, which suggests that Rho may play a role in preventing rejection after heart transplantation.

### 3.5. Rho Regulates the Immune Function of DCs by Affecting Mitochondrial Dynamics

**Figure 6:**
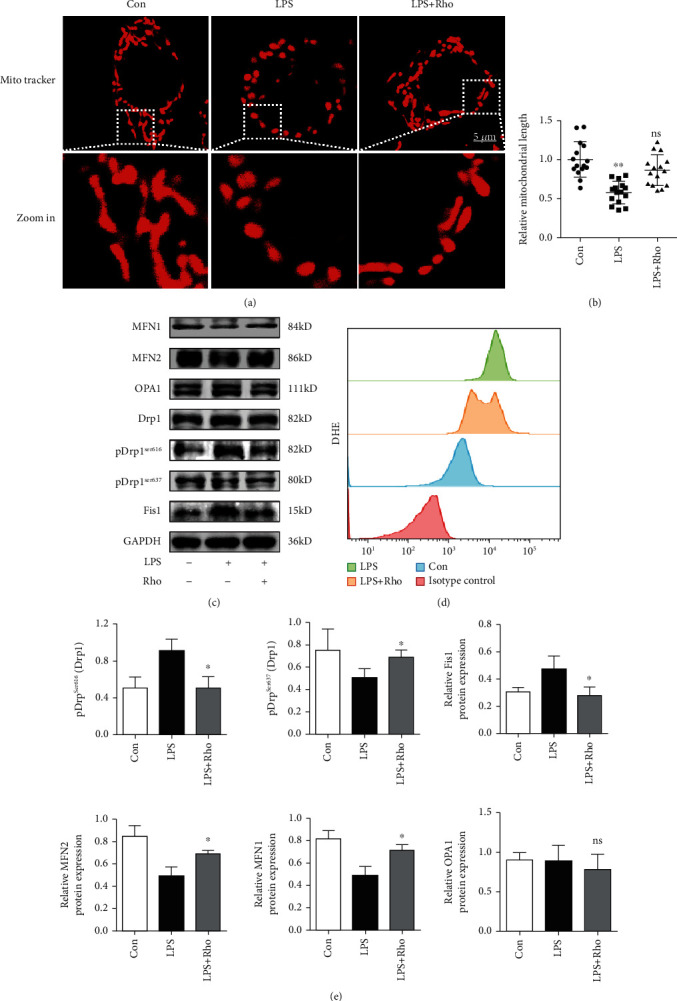
Rho inhibits the maturation of DCs by promoting mitochondrial fusion activity. (a) BMDCs were stained with MitoTracker (mitochondria, red). LPS stimulation clearly promoted mitochondrial fragmentation. The mitochondria morphology of Rho group showed into the mitochondrial fusion. (b) The relative mitochondrial length of different groups. (c) The expressions levels of mitochondrial fusion- and fission-related proteins in different groups of DCs were determined by Western blot assays. (d) The ROS production by DHE assays. (e) The protein expressions were quantified by ImageJ. The data are representative images or expressed as the mean ± SEM of three independent experiments. ^∗^*P* < 0.05, ^∗∗^*P* < 0.01 versus the LPS group.

We further investigated the mechanisms underlying Rho-mediated induction of immature DCs. When DCs transform from relative quiescence to high activity, changes in mitochondrial dynamics have important effects on the functional status of immune cells. Mitochondria, which supply energy to cells, are usually short rods or spheroids, and many mitochondria form a network structure. Mitochondrial fusion and division can damage this network structure. Confocal microscopy showed that mitochondrial fission was promoted during the maturation process of DCs, providing more energy to support DCs in their antigen presentation role. Mitochondrial dynamics include fusion, fission, transport, and mitophagy [34]. Previous studies have shown that the state of mitochondrial fusion and fission affects the immune response. For example, Stat2-Drp1-mediated mitochondrial fission is necessary for the proinflammatory differentiation of macrophages [24]; the mitochondrial fusion protein Opa1 can determine the fate of memory T cells but not Teff cells [23]. There are some reports showing that Rho can inhibit mitochondrial fission and ROS production in mitochondria [35, 36]. To verify that Rho can inhibit the maturation of BMDCs by mediating mitochondrial dynamics, we first examined mitochondrial morphology in immature DCs, mature DCs, and Rho-treated DCs. Confocal microscopy showed that mature DCs significantly had shorter mitochondria than immature DCs (Figures 6(a) and 6(b)), and Rho-treated DCs exhibited mitochondrial fusion. Therefore, we evaluated the expression of proteins involved in mitochondrial fusion and fission. The results showed that Rho enhanced the expression of the mitochondrial fusion-related proteins MFN1 and MFN2 and decreased the expression of the mitochondrial fission-related factors Drp1 and Fis1 (Figures 6(c) and 6(e)). However, the fusion protein OPA1 did not show differences between the different groups. We hypothesize that mitochondrial fusion mainly occurred in the outer mitochondrial membrane. Since inhibiting ROS generation may inhibit DC differentiation [37], the ROS production in BMDCs was evaluated using a DHE assay, and the results showed that Rho treatment decreased ROS generation (Figure 6(d)). Meanwhile, the mitochondrial ROS was detected, and the results were shown in Supplementary Figure S6, which suggested that Rho reduced the production of not only intracellular ROS but also mitochondrial ROS.

### 3.6. Rho Promotes Mitochondrial Fusion Mainly through MFN1

**Figure 7:**
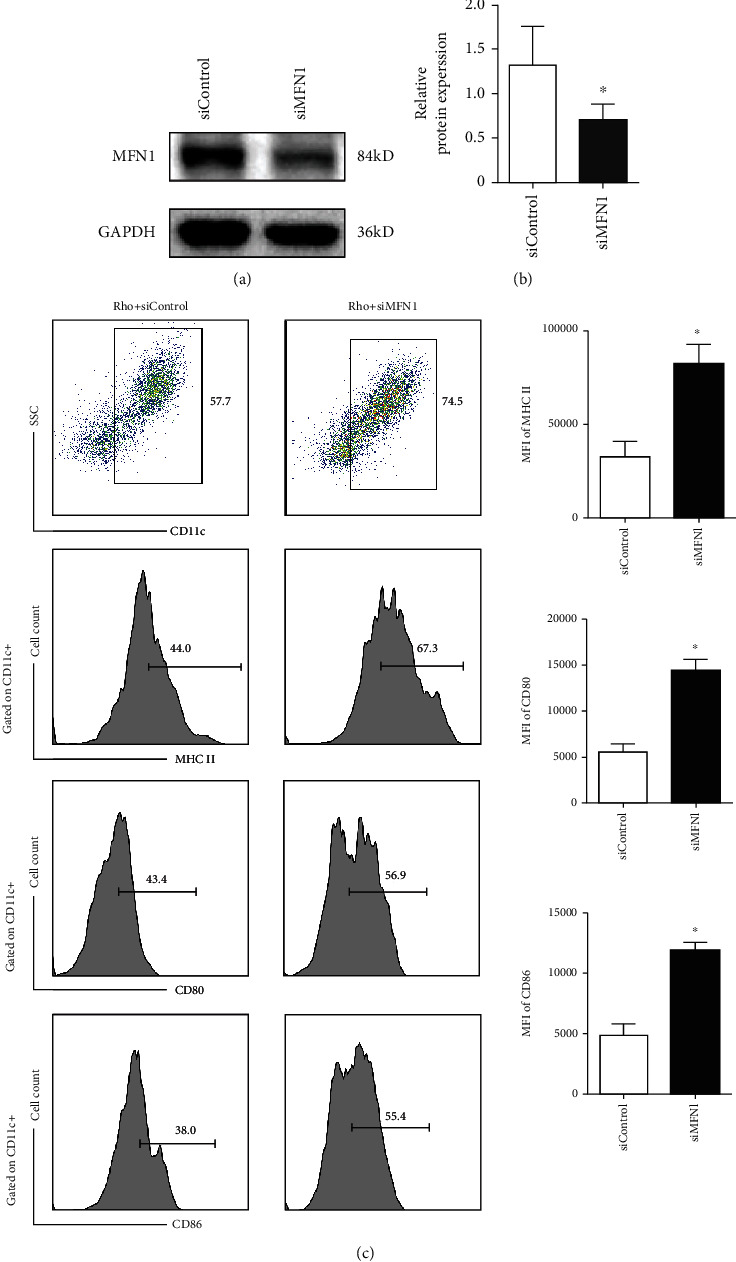
Rho hinders the BMDCs maturation mainly by the mitochondrial fusion protein MFN1. (a) The knockdown result of the MFN1 expression in BMDCS transfected using siMFN1. (b) The relative protein expression analysis by ImageJ after MFN1 knockdown. (c) Flow cytometry analysis of CD80, CD86, and MHC II in CD11c population in the Rho + siControl group and Rho + siMFN1 group. The data are representative images or expressed as the mean ± SEM of three independent experiments. ^∗^*P* < 0.05, ^∗∗^*P* < 0.01 versus the Rho + siControl group.

To determine whether mitochondrial fusion is required for Rho-mediated inhibition of BMDCs, we silenced MFN1 using a selective small interfering RNA. BMDCs were treated for 48 h with either control siRNA or MFN1-specific siRNA. The MFN1 knockdown (with siRNA) results are shown in Figures 7(a) and 7(b). The expression levels of MHC II, CD80, and CD86, which represent the maturation states of BMDCs, were then measured by flow cytometry. High expression of CD80, CD86, and MHC II in the CD11c subset suggests that MFN1 knockdown can effectively reverse Rho-mediated inhibition of DC maturation (Figure 7(c)). These findings suggest Rho does not inhibit mitochondrial fusion during the maturation of DCs. These results further suggest that mitochondrial fusion participates in the maturation and/or activation of Rho-treated DCs. Our findings suggest that Rho can induce quiescence in DCs, which is mainly mediated by mitochondrial fusion.

## 4. Discussion

Cardiac transplantation is an effective treatment for cardiac failure, particularly for those patients who are resistant to aggressive medical therapy. However, acute allograft rejection is still a challenge for patients who undergo transplantation. Acute rejection after heart transplantation is widely present in posttransplant patients, and the acute transplant rejection plays an important role in patient prognosis. Thus, it is extremely important to develop new drugs to treat acute transplant rejection. In the present study, the combination of Rho and MMF significantly attenuated acute transplant rejection, as evidenced by the reduced immune infiltration and apoptosis in cardiac myocytes. We also found decreased CD4^+^ Teff cells and increased Tregs in the spleen and LNs of transplanted mice, and the attenuation of DC maturation was observed in vivo and in vitro. DCs are central in acute transplant rejection and are upstream cells that induce maturation in various types of immune cells. We found that the combined treatment could inhibit DC maturation and attenuate rejection by upregulating MFN1, a mitochondrial fusion-related protein.

MMF is a prodrug of mycophenolic acid (MPA), an inhibitor of inosine monophosphate dehydrogenase (IMPDH) which is a key enzyme the de novo synthetic pathway of guanine nucleotides; therefore, MMF can inhibit the proliferation of B and T lymphocytes. MMF is frequently used as an antimetabolite in the treatment of acute transplant rejection, but the side effects of MMF limit its use in large quantities and for long periods of time. Based on our previous studies, natural phytomedicinal monomers are capable of complementing and mitigating the doses of traditional immunosuppressants [28]. However, MMF as a posttransplantation immunosuppressant is not common at present. To promote the use of MMF in cardiac transplantation, we compared the efficacy of MMF with the more common immunosuppressant FK506 in cardiac transplantation and showed that both were protective in cardiac transplantation and that there was no statistical difference in the therapeutic effect. The experimental results are supplemented in Supplementary Figure S7-S10. Some natural monomers derived from medical plants have been shown to have cardioprotective effects [38, 39]. Rho is a natural phytomedicinal monomer that has been shown to have antioxidant, anti-inflammatory, and antitumor effects and can be used to treat numerous diseases [21, 40]. According to the very convincing references, the Rho has a high level of biosafety. A lot of studies on efficacy of Rhodosin have mainly focused on cell lines, mice, and drosophila. There was a phase II randomized placebo controlled clinical trial proved that Rhodosin produced less antidepressant effect versus sertraline but also resulted in significantly fewer adverse events and was better tolerated [41]. The Ames test and chromosomal aberration of CHO cells and mouse marrow micronucleus assay were used to evaluate the genetic toxicity of Rhodosin. The results indicate that Rhodosin injection has no genetic toxicity [42]. Another study also proved the safety of Rhodosin in genomics [43]. And a development toxicity study of Rhodosin injection in SD rats showed that Rhodosin does not have conspicuous parent toxicity, teratogenic effect, and embryotoxicity in SD rat even with the dose of 0.5 g/kg [44]. Taking into account the results of these clinical human studies and the extensive animal studies, we can demonstrate that Rho has a high biosafety profile.

We showed that Rho can regulate immune cell status. Therefore, the immunosuppressive roles of MMF and Rho are in different stages of acute rejection response, which are not conflicting, but rather harmonizing and cooperating. Based on these advantages, combined treatment was able to reduce the dose of MMF and prolong the survival of mice after heart transplantation.

DCs connect innate with adaptive immunity and are sentinel cells in the development of graft rejection. The costimulatory molecules such as MHC II, CD80, and CD86 expressed by imDC were kept at very low levels. Meanwhile, the imDCs do not overexpress proinflammatory factors such as IL-1*β*, IL-6, and IL-12 but rather promote the expression of anti-inflammatory factors such as IL-10 and TGF-*β*; based on this mechanism, the imDCs could induce T cell anergy and Treg responses [45–47]. Immature DCs have been shown to mitigate rejection in heart transplantation, and our in vivo and in vitro experiments showed that Rho inhibited DC maturation and that Rho-treated DCs suppressed T cell activation, which explains why Rho-treated DCs exert an immunosuppressive effect.

Energy metabolism plays a vital effect in the functional state of immune cells [48]. It has been experimentally demonstrated that the maturation state of DCs is related to their intracellular energy metabolism [49]. As the only energy-supplying organelle within cells, mitochondria play an extremely important role in energy metabolism, and mitochondrial dynamics are critical for regulating the functional state of mitochondria [50]. In our study, Rho inhibited mitochondrial fission and promoted mitochondrial fusion in DCs. Among the proteins associated with mitochondrial fusion, the western blot results showed that MFN1 was significantly altered and that changes in mitochondrial functional were accompanied by changes in ROS levels through a mutually influential and reciprocal feedback relationship.

MFN1, located in the outer mitochondrial membrane, is a protein that regulates mitochondrial fusion, and our study proved that MFN1 was significantly altered by the Rho treatment. To test the mechanism by which Rho inhibits DC maturation, we successfully knocked down MFN1 in DCs using si-MFN1. When MFN1 was knocked down, the inhibitory effect of Rho on DC maturation was partially reversed. Rho has been shown in other studies to promote mitochondrial fusion in cells by increasing MFN1 expression, and our findings are consistent with these studies. We proved for the first time that were was a relationship between DC maturation and mitochondrial dynamics.

Our experiments provide an alternative for clinical immunosuppressive therapies, and our experiments show the relationship between mitochondrial dynamics and immune responses, providing new ideas for the development of immunosuppressive agents. But there is a limitation in this study, since the mitochondrial dynamics have close relationship with ischemia-reperfusion injury; we believe that the use of Rho may reduce ischemia-reperfusion injury after transplantation. In contrast, ischemia-reperfusion injury is also an important cause of graft immune rejection, and we need to further explore the role of Rho in antioxidant and anti-ischemia-reperfusion injury, which will combine the antioxidant and immunosuppressive effects of Rho.

**Figure 8:**
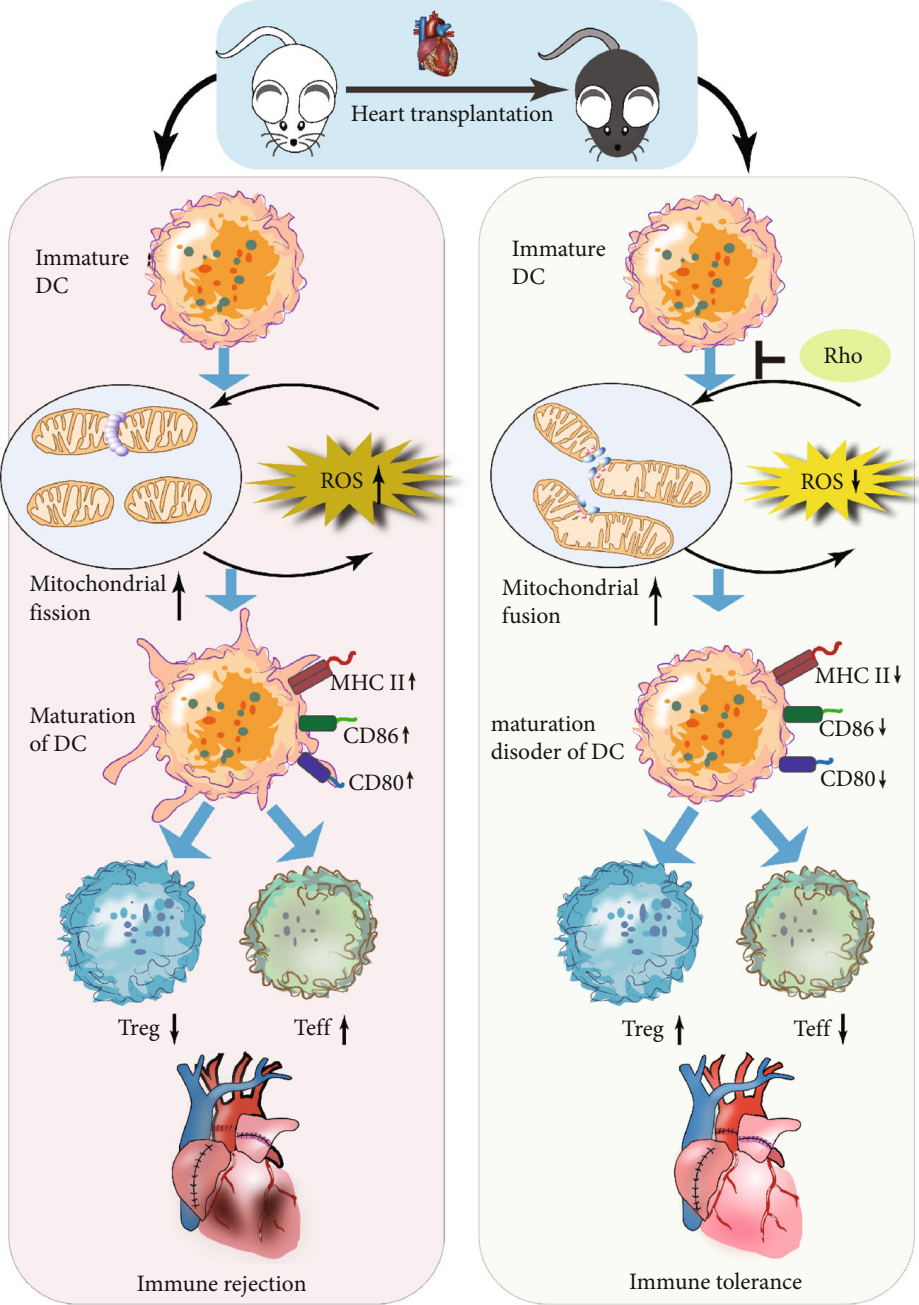
Diagram of Rho preventing from acute rejection in heart transplantation. In MHC II-full mismatch cardiac transplantation, we proved that combination of Rho and MMF prolongs cardiac transplantation survival, and Rho can promote the mitochondrial fusion mainly by MFN1 and decrease the level of ROS, then induce tolerogenic DCs which can suppress the Teff population and increases the Treg, so the Rho plays an immune tolerant effect in murine heart transplantation.

Our data suggest that the combination of Rho and MMF extends the survival time of transplanted hearts and that Rho plays an important role in inhibiting DC maturation by modulating mitochondrial dynamics. The molecular mechanism of Rho in preventing acute rejection in heart transplantation is presented in Figure 8. Rho could be a potential clinical adjunct to aid in the use of immunosuppressive drugs and reduce the toxicity and side effects due to the amount of antirejection drugs used. Since no detectable side effects of Rho were observed and Rho is clinically used for heart protection, Rho has promising clinical applications in organ transplantation as a novel immunosuppressant. Overall, these properties of Rho and the results obtained above proved the clinical significance of this study. Our study provides an efficient strategy to alleviate acute rejection after organ transplantation effectively and safely.

## Data Availability

The data used to support the findings of this study are available from the corresponding author upon request.

## Abbreviations

Rho: Rhodosin
MMF: Mycophenolate mofetil
DCs: Dendritic cells
Tregs: Regulatory cells
MHC: Major histocompatibility complex
BMDCs: Bone marrow-derived dendritic cells
imDCs: Immature dendritic cells
mDCs: Mature dendritic cells
GM-CSF: Recombinant granulocyte macrophage colony-stimulating factor
dLNs: Draining lymph nodes
Teff: Effector T cell
MFN1: Mitofusin 1
MFN2: Mitofusin 2
OPA1: Optic atrophy 1
Fis1: Fission protein 1
Drp1: Dynamic-related protein 1
qRT-PCR: Quantitative real-time polymerase chain reaction
IL: Interleukin
TNF-*α*: Tumor necrosis factor
CFSE: Carboxyfluorescein diacetate succinimidyl ester
SEM: Scanning electron microscopy images

## Supplementary Materials

Supplementary Materials Supplementary Materials Figure S1: the expressions of the inflammatory factors in transplanted hearts. Figure S2: the ratio of CD4 Teff/Treg in spleens and LNs harvested from allotransplantation. Figure S3: the SEM (scanning electron microscopy) images of immature DC, mature DC, and Rho-treated DC. Figure S4: the immunofluorescence staining of MHC II of BMDCs in different groups. Figure S5: (a) Results of CD4^+^ T cells sorting and purification. (b) FACS-sorted CD4^+^ T cells from naïve C57BL/6 mice were labeled with CFSE and costimulated with Rho or without Rho for 72 h. (c) The frequency of CD4^+^IL10^+^ T cells in different groups. (d) The frequency of CD4^+^IL-17^+^ T cells in different groups. Figure S6: the MitoROS result in different groups. Figure S7–S10: comparison of the efficacy of MMF with FK506 in cardiac transplantation.
