# Supplementary material for: The Combination of Rhodosin and MMF Prolongs Cardiac Allograft Survival by Inhibiting DC Maturation by Promoting Mitochondrial Fusion
Source: Oxid Med Cell Longev. 2022 Jul 9;2022:7260305. doi: 10.1155/2022/7260305 (PMC9288296; doi:10.1155/2022/7260305)
Supplement: Supplementary Materials — Figure S1: the expressions of the inflammatory factors in transplanted hearts. Figure S2: the ratio of CD4 Teff/Treg in spleens and LNs harvested from allotransplantation. Figure S3: the SEM (scanning electron microscopy) images of immature DC, mature DC, and Rho-treated DC. Figure S4: the immunofluorescence staining of MHC II of BMDCs in different groups. Figure S5: (a) Results of CD4+ T cells sorting and purification. (b) FACS-sorted CD4+ T cells from naïve C57BL/6 mice were labeled with CFSE and costimulated with Rho or without Rho for 72 h. (c) The frequency of CD4+IL10+ T cells in different groups. (d) The frequency of CD4+IL-17+ T cells in different groups. Figure S6: the MitoROS result in different groups. Figure S7–S10: comparison of the efficacy of MMF with FK506 in cardiac transplantation. [file 7260305.f1.pdf]

Supplementary material

The combination of Rhodosin and MMF prolongs cardiac allograft survival by inhibiting DC maturation by promoting mitochondrial fusion

Yanjia Che [1,2,3,†], Yuanyang Chen[1,2,3,†], Zhiwei Wang[1,3,*] , Sihao Zheng [1,2,3,†], Kai Xing[1,2,3], Shun Yuan[1,2,3], Xiaohan Zhong[1,2,3]

[1] Department of Cardiovascular Surgery, Renmin Hospital of Wuhan University, Wuhan 430060，China

[2] Cardiovascular Surgery Laboratory, Renmin Hospital of Wuhan University, Wuhan 430060, China

[3] Central Laboratory, Renmin Hospital of Wuhan University, Wuhan,430060, China

* Correspondence: wangzhiwei@whu.edu.cn

†These authors contributed equally to this work.

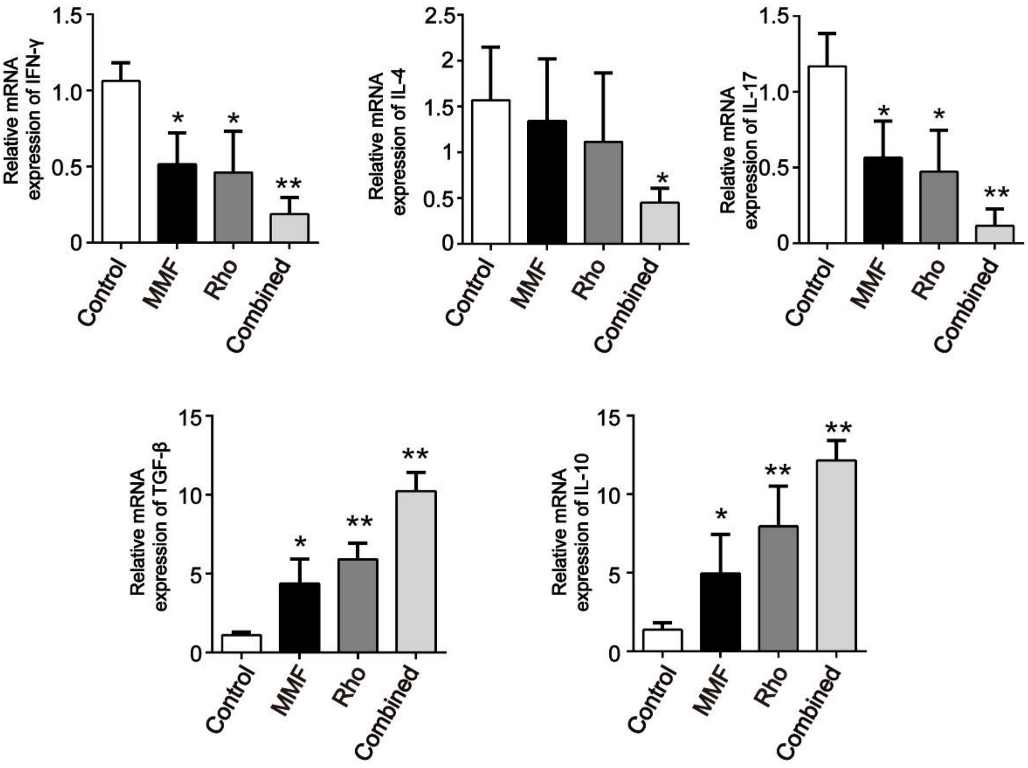

Supplementary FIGURE 1: The expressions of the inflammatory factors in transplanted hearts. Elevated expression of anti-inflammatory IL-10 and TGF-β were tested in combination administration group. And reduced levels of pro-inflammatory factor include IL-4, IL-17 and IFN-γ expression in the combination administration group was observed.

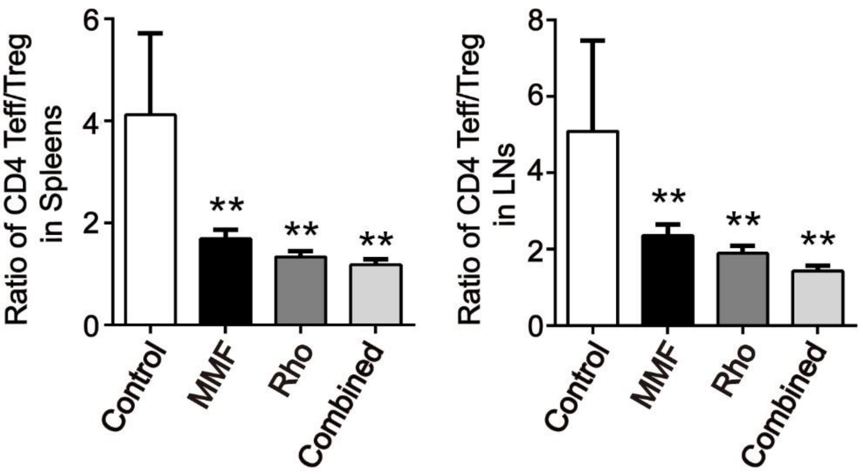

Supplementary FIGURE 2: The ratio of CD4 Teff/Treg in spleens and LNs harvested from allotransplantation.

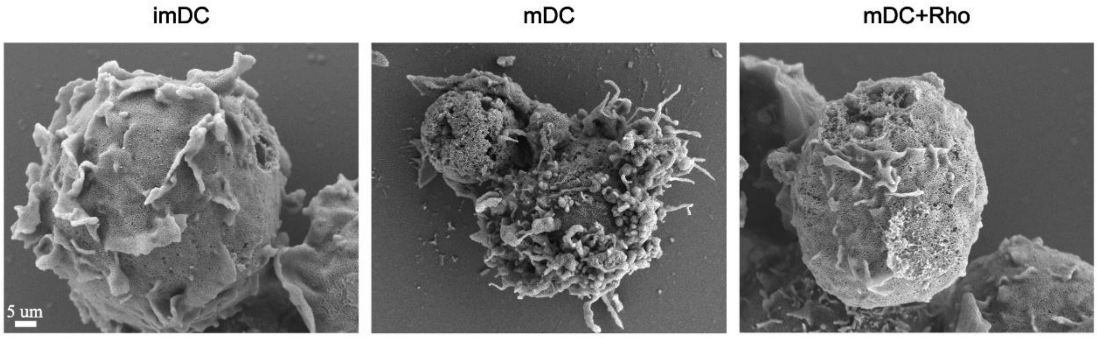

Supplementary FIGURE 3: The SEM (Scanning Electron Microscopy) images of immature DC, mature DC and Rho treated DC.

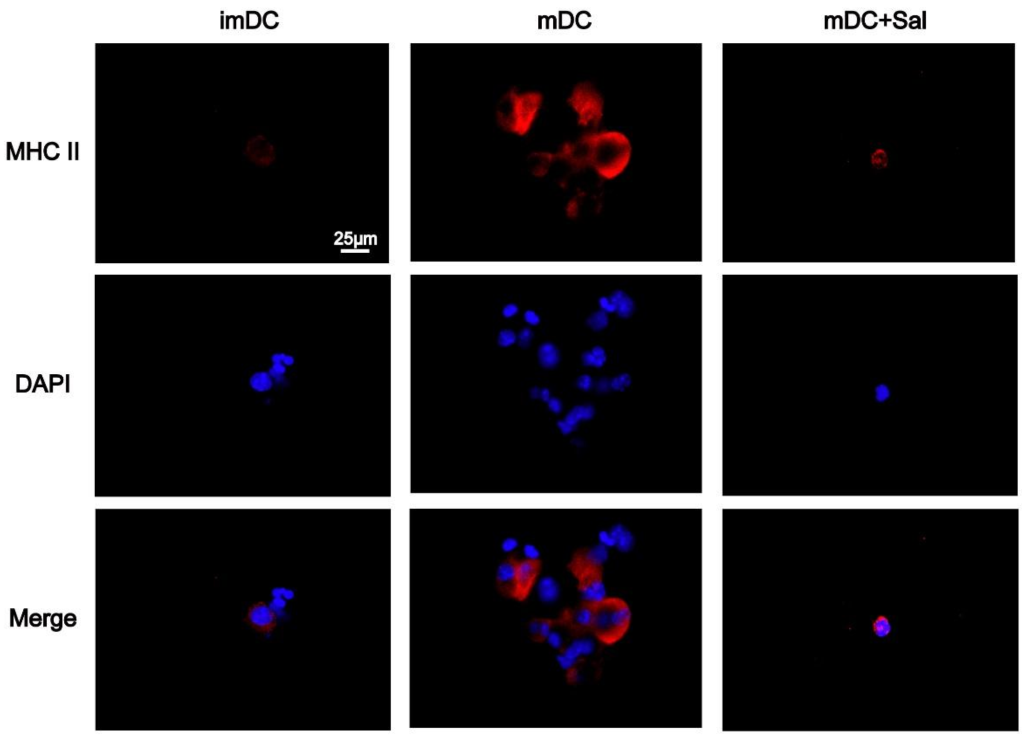

Supplementary FIGURE 4: The immunofluorescence staining of MHC II of BMDCs in different groups.

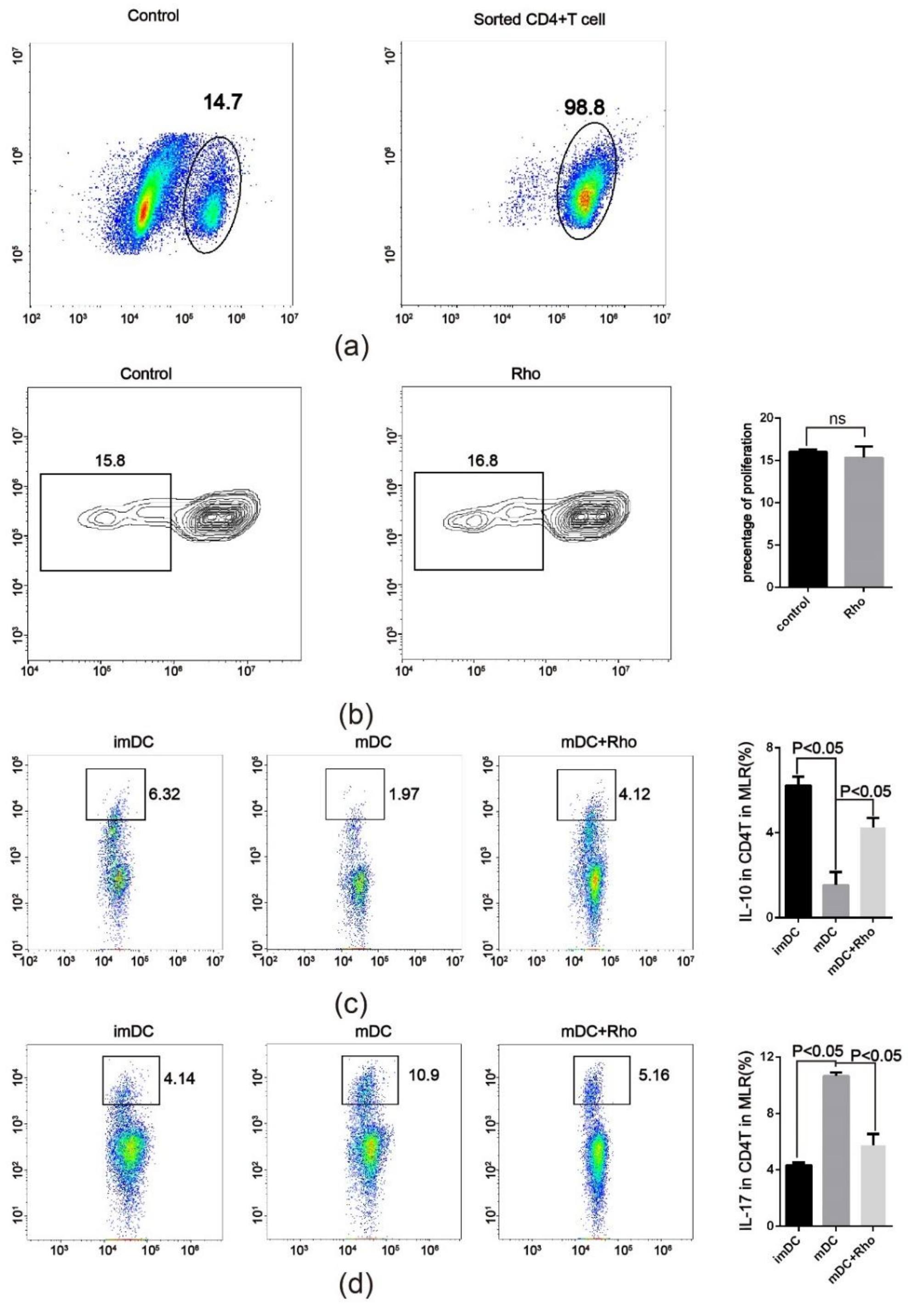

Supplementary FIGURE 5: (a) Results of CD4+T cells sorting and purification. (b) FACS-sorted CD4+ T cells from naïve C57BL/6 mice were labeled with CFSE and co-stimulated with Rho or without Rho for 72h. (c) The frequency of CD4+IL10+ T cells in different groups. (d) The frequency of CD4+IL-17+ T cells in different

groups.

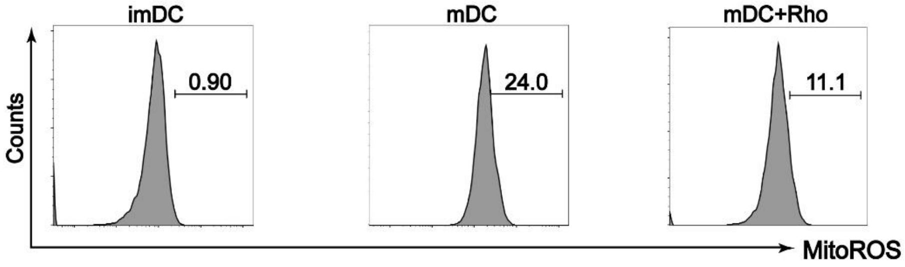

(a)

Supplementary FIGURE 6: The MitoROS result in different groups.

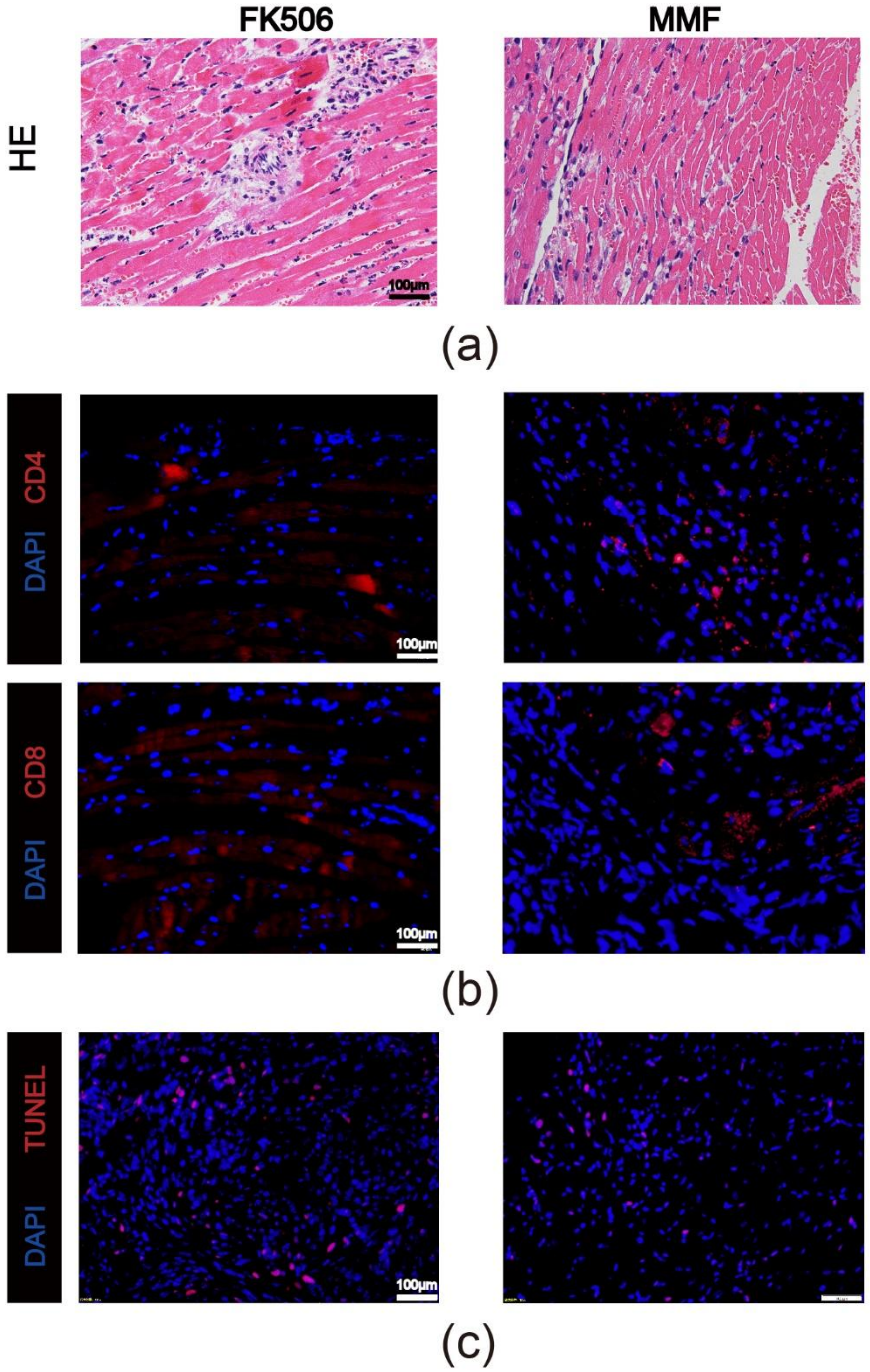

Supplementary FIGURE 7: (a) Hematoxylin-eosin (HE) staining on the cardiac allografts from FK506 group and MMF group. (b) The infiltration of CD4+T cells and CD8+T cells in transplanted hearts from FK506 group and MMF group. (c) The immunofluorescence staining of TUNEL in allografts from FK506 group and MMF group.

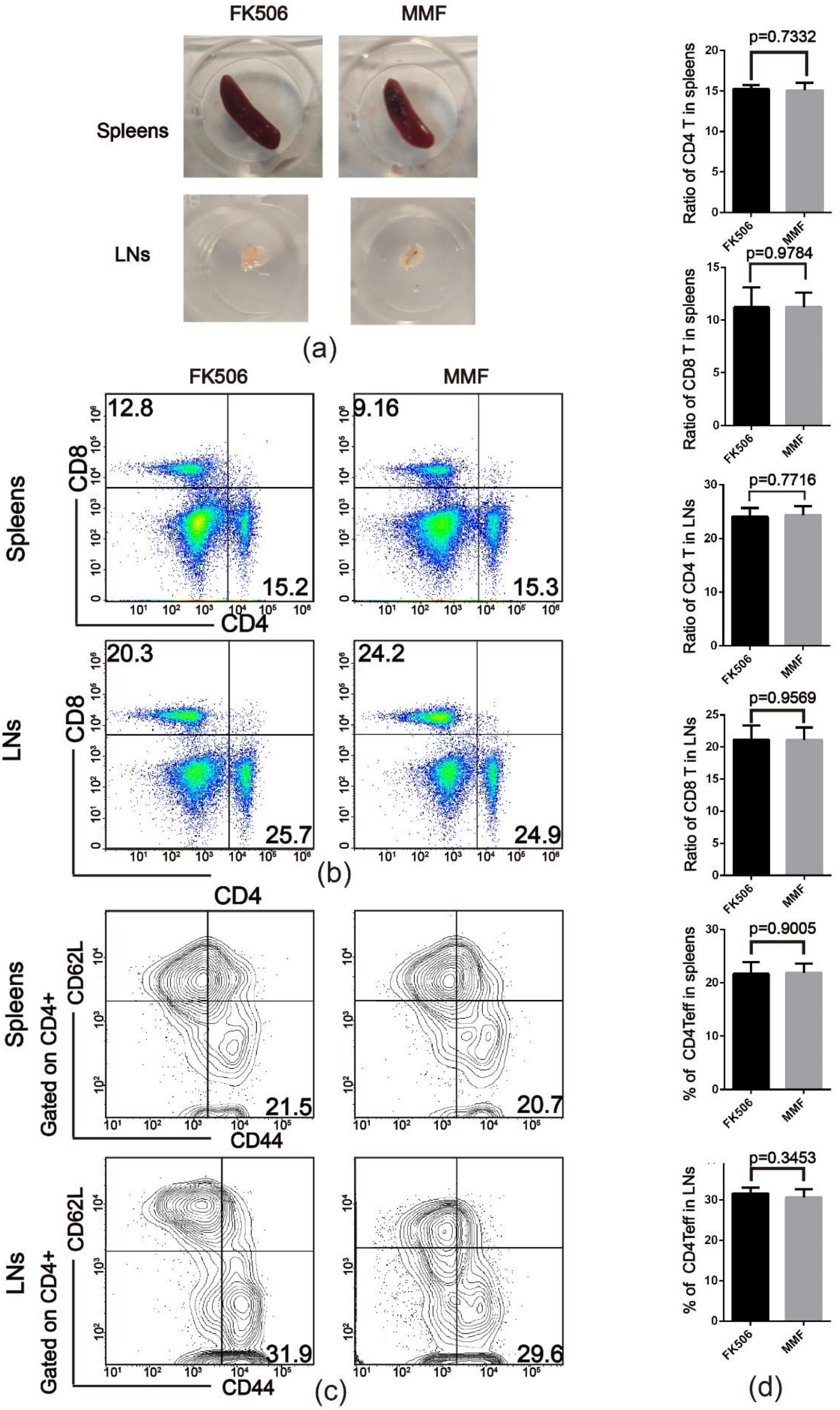
FK506
MMF
Spleens
LNs
(a)
FK506
MMF
12.8
9.16
CD8
15.2
15.3
CD4
Spleens
20.3
24.2
LNs
25.7
24.9
(b)
CD4
CD62L
Spleens
Gated on CD4+
21.5
20.7
CD44
LNs
31.9
29.6
(c)
p=0.7332
Ratio of CD4 T in spleens
p=0.9784
Ratio of CD8 T in spleens
p=0.7716
Ratio of CD4 T in LNs
p=0.9569
Ratio of CD8 T in LNs
p=0.9005
% of CD4Teff in spleens
p=0.3453
% of CD4Teff in LNs
(d)

Supplementary FIGURE 8: Effect of FK506 and MMF on the quantities of CD4+ and CD8+ T cells. The proportions of CD4+ and CD8+ T cells in the spleens and draining lymph nodes were determined by flow cytometry on Day 5 after heart transplantation (n=6 mice per group). (a) Macroscopic appearances of spleens and lymph nodes isolated from FK506 group and MMF group, Rho group and combination treatment group on Day 5 after transplantation. (b) The proportions of CD4+ and CD8+T cells in the spleens and dLNs were determined by flow cytometry on Day 5 after transplantation (n=6 mice per group). (c) The frequency of CD4+T effector cells (CD4+CD44^{hi}CD62L^{low}) in spleens and draining lymph nodes in FK506 group and MMF group. (d) The quantitative analysis of the CD4+ T, CD8+ T, CD4+Teff cells in spleens and draining lymph nodes by GraphPad Prism 8.0. Data of histograms are presented as means ± SD from six independent experiments (*P<0.05, **P<0.01, n=6 mice MMF group versus FK506 group).

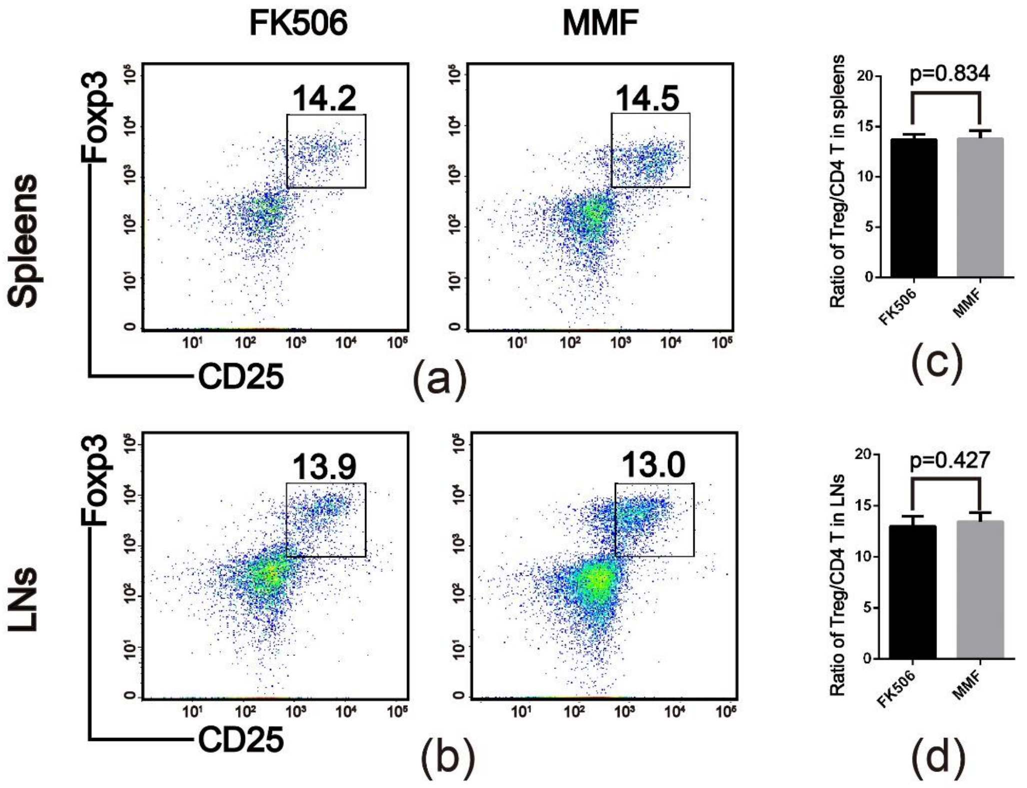

Supplementary FIGURE 9: (a) Percentage of Treg (CD4+CD25+Foxp3+) cells in spleens derived from the FK506 group and MMF group. (b) Percentage of Treg (CD4+CD25+Foxp3+) cells in dLNs derived from the FK506 group and MMF group (c) (d) There was no statistical difference in the quantities of Treg of spleens and dLNs between the MMF group and FK506 group.

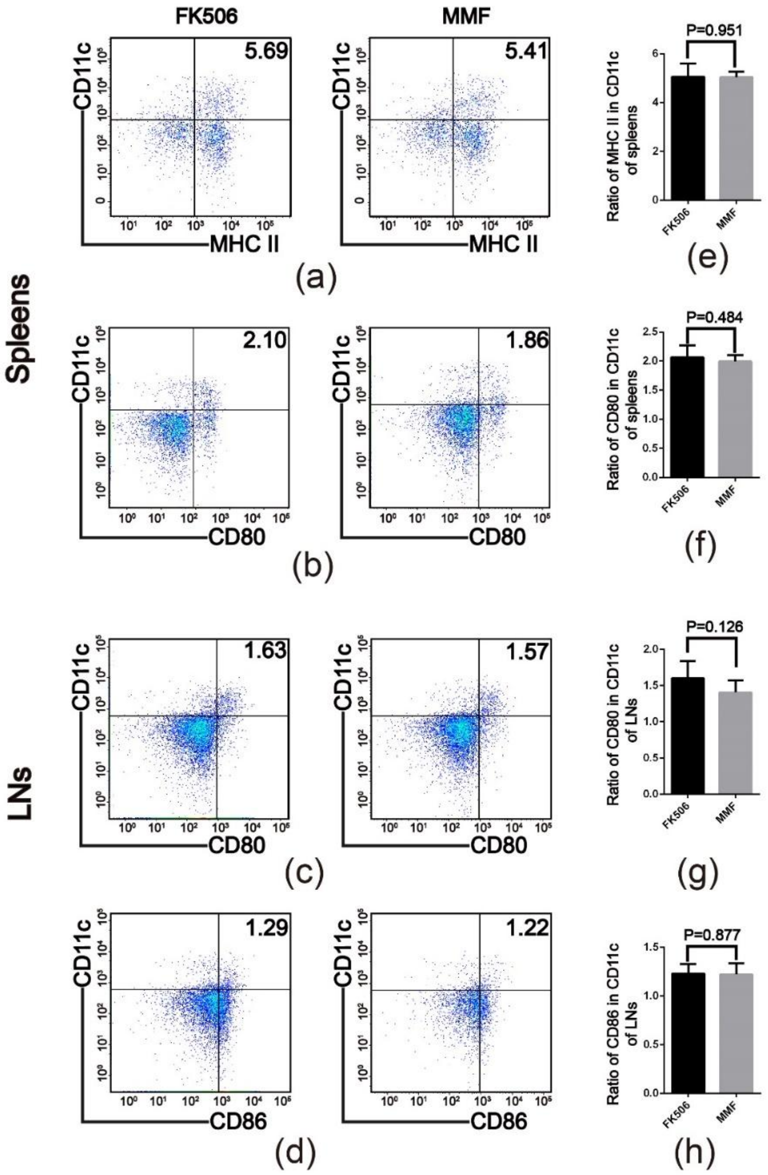

Supplementary FIGURE 10: There is no statistical difference in the DCs maturation *in vivo* between the MMF group and FK506 group. (a)-(d) The percentage of CD86+ MHC II+ CD80+ DCs within CD11c+ population were determined via FACS analysis. Data of histograms are presented as means ± SD from six independent experiments (*P<0.05, **P<0.01, n=6 mice per group FK506 group versus MMF group). (e)-(h) The quantitative analysis of percentage of CD86+ MHC II+ CD80+ DCs within CD11c+ population in spleens and draining lymph nodes by GraphPad Prism 8.0. Data of histograms are presented as means ± SD from six independent experiments (*P<0.05, **P<0.01, n=6 mice FK506 group versus MMF group).
